# Cervical cancer screening in Sweden 2014-2016

**DOI:** 10.1371/journal.pone.0209003

**Published:** 2018-12-17

**Authors:** Maria Hortlund, K. Miriam Elfström, Pär Sparén, Pouran Almstedt, Björn Strander, Joakim Dillner

**Affiliations:** 1 Department of Laboratory Medicine, Karolinska Institutet, Stockholm, Sweden; 2 Department of Medical Epidemiology and Biostatistics, Karolinska Institutet, Stockholm, Sweden; 3 Swedish Cervical Screening Registry, Regional Cancer Center West, Gothenburg, Sweden; 4 Department of Obstetrics and Gynecology, Institute of Clinical Science, Sahlgrenska Academy, University of Gothenburg, Gothenburg, Sweden; 5 Swedish National Cervical Screening Registry, Center for Cervical Cancer Prevention, Department of Pathology, Karolinska University Laboratory, Karolinska Hospital, Stockholm, Sweden; University of Zurich, SWITZERLAND

## Abstract

**Background:**

To enable incremental optimization of screening, regular reporting of quality indicators is required.

**Aim:**

To report key quality indicators and basic statistics about cervical screening in Sweden.

**Methods:**

We collected individual level data on all cervical cytologies, histopathologies, human papillomavirus tests and all invitations for cervical screening in Sweden during 2013–2016.

**Results:**

There were over 2,278,000 cervical samples collected in Sweden in 2014–2016. Organized samples (resulting from an invitation) constituted 69% of samples. The screening test coverage of all resident women aged 23–60 was 82%. The coverage has slowly increased for >10 years. There is large variability between counties (from 71% to 92%) over time. There were 25,725 women with high-grade lesions in cytology during 2013–2015. Only 96% of these women had a follow-up histopathology within a year. Cervical cancer incidence showed an increasing trend.

**Conclusion:**

Key quality indicators such as population coverage and follow-up rates were stable or improving, but there was nevertheless an unexplained cervical cancer increase.

## Introduction

Cervical screening is a globally recommended public health policy, which is in place in most European countries but in varied formats [1]. Evidence-based surveillance and quality assurance of a screening programme to achieve high population coverage and high cancer-preventive effect uses cervical screening registries. Even though most EU countries have cervical screening registries and quality assurance programs in place, the registries and the results of their work is commonly not available in the English language [1]. There is a growing awareness that international reporting is necessary for international exchange of experiences, promoting progress. For example, the Danish national cervical cancer screening registry published how the registry is used for follow-up and research as well as the results of nine key quality indicators [2]. The Swedish National Cervical Screening Registry (NKCx) monitors and evaluates the extent, quality and effect of screening, based on reports of all screening invitations, cervical cytologies, histopathologies, and human papillomavirus (HPV) tests in the country. Key quality indicators (e.g. population test coverage, diagnostic profiles, population coverage of invitations, and proportion of women with abnormal tests that are followed up) and basic statistics are reported back to the organized cervical screening programs in each region [3]. We report here how we use the collection of individual-level input data to calculate quality indicators for cervical screening in Sweden.

## Materials and methods

The setting is Sweden, where the nationally mandated program has hitherto included that all resident women should receive an invitation by letter for cytology screening at a specified time and place (usually at a Maternity Care Center in the vicinity of the residential address of the woman). The invitations are sent at 3-yearly intervals 23–49 year of age and at 5-yearly intervals 50–60 years of age. In June 2015, the national program was changed to a mostly HPV-based screening program, but the change had not been implemented immediately (was implemented during 2017 and 2018). For issuing invitations, the population registry is used for identification of resident women. Linkage with files from the cytology/pathology/microbiology laboratories will then assess if a cervical sample has already been taken in the recommended interval, in which case no invitation is issued (“sorting out”). Women who do not attend their appointment will remain in the pool of resident women, as no sample was taken, and will have a new invitation with a new appointment issued next year.

Data collection and data analysis was performed as described [3]. Briefly, all laboratories in Sweden that perform cervical cytology, cervical histopathology (26 laboratories), and HPV testing (28 laboratories) and all units that issues invitations for screening (22 units. Usually the laboratory is also the unit that issues the invitation) are every year asked to export a file with individual level data (subject ID, sample ID, sampling date, diagnostic codes on analysis results or for invitations subject id, date of invitation issue and, appointment date) to a cervical screening registry. The registry has in its current form been in operation since 2012. An early version of the registry was launched in the mid-1990:ies, primarily in order to be able to follow up a nationwide randomized HPV screening trial [4]. The different imports are linked using the unique personal identifier (subject id) that is assigned to all citizens at birth or immigration. As all laboratories that perform tests and all units that issue invitations participate in the program and all of them export a copy of the same information as is sent to the women or reported to the laboratory customers, the registry is essentially 100% complete. All citizens are entitled to withdraw, but so far only 1 woman has withdrawn from the registry. The registry thus contains all data from all women in Sweden, except 1 person.

Population test coverage is calculated as the number of women in the age group under study who were resident in a county (or the country) that has had a cervical sample taken in the preceding 3 or 5 years, respectively, divided by the total number of women in the age group under study who were resident in the county during this time. Data on smears taken are imported from laboratories as described above and data on resident women is imported from the population registry, maintained at the Swedish Tax Office.

Proportion of smears in the organized screening is estimated as the number of smears flagged by the laboratories to be organized smears divided by the total number of smears. For one county that did not do these flagging, smears taken at maternity care centers known to be screening stations for organized screening were considered to be organized smears.

Attendance rate after invitation was calculated as the inverse of the survival function (1-probability not to participate) by the Kaplan-Meier method. The table shows the cumulative proportion of women who have had a screening test following an invitation.

The cumulative proportion of high-grade squamous intraepithelial lesions (HSIL) and adenocarcinoma in situ (AIS) in cytology that were followed with a biopsy anywhere in the country, within 3 month and within 1 year, were calculated as the inverse of the survival function (1-probability of not having a biopsy) by using Kaplan-Meier method. Delay of follow-up with biopsy beyond 3 months is known to increase the risk for cancer [5]. As entirely missing follow-up increases the cancer risk even more than a delayed follow-up [5], the proportion of women followed up by 12 months is also reported.

Sweden reports cytology and histopathology results using the Standardized Nomenclature for Medical Diagnoses (SNOMED), but there is still a plethora of non-standard codes in use. In 2013, there were 2,442 different codes reported that could not be interpreted. Since then systematic inquiries have been sent to the laboratories each year, which have resulted in that during 2014, 2015 and 2016, there were only 21, 17, and 38 non-interpretable diagnostic codes (non-interpretable codes are retained in the database as a separate category of results).

The proportion and number of smears with different cytological diagnoses by laboratory (the “diagnostic profile”) is published at www.nkcx.se, with the intent to highlight possible differences in diagnostic practices.

The collection of individual level data from all women in Sweden was approved by the Ethical Review Board of Stockholm, Sweden (2011/1026-31/4), which decided that individual informed consent was not required. In Sweden, ethical review boards are appointed by government, chaired by a senior judge and have the authority to determine requirements for consent.

Data on incidence of cervical cancer was obtained from the Swedish National Cancer registry (www.socialstyrelsen.se) also available in English at www-dep.iarc.fr/NORDCAN and presented here for comparative purposes only. All data in this paper is available at the Swedish National Cervical Screening Registry at www.nkcx.se. Presentations of the data are also available at www.nordscreen.org. This data was collected by us as described in this paper and by Elfström et. al [3]. The authors also have access to personal identities of the individuals in the registry. However, this paper does not present any personally identifiable information at all. Trends in incidence were analyzed using the Wald test for trend and Poisson regression using Statistical Analysis Software (SAS V9.4, SAS Institute, Cary, North Carolina, USA).

## Results

The number of screening tests in Sweden varied from 723,500, 778,621 and 776,011 during 2014, 2015 and 2016. From the target age groups 23–60 years there were 662,350, 695,648 and 702,946 screening tests collected in 2014, 2015 and 2016, respectively (Table 1). The number of HPV tests increased from 28,803 in 2013, to 79,688 in 2014, 137,300 in 2015 and 156,683 in 2016. Overall, 69% of tests were organized (resulted from personal invitations). The other 31% of tests include spontaneous testing and follow-up smears taken after referrals from the organized program. Participation within 3 months following an invitation was 55%, 68% and 57% in each year (Table 2). Many women change the appointment time in the invitation and participation 12 months after invitation is also reported (68%). Population test coverage in the age range targeted for screening was stable at 81–82%, varying from 69 to 92% in different counties and years (Table 1). Changes in population test coverage were not systematically evaluated for statistical significance. The denominators were large, resulting in that most changes were statistically significant. For example, in a medium-sized county with a target population of 63000 women (Södermanland) changes in population test coverage of >0,5% are significant at the p<0,05 level (Chi-square test).

**Table 1:** Number of cervical smears, proportion of organized smears, and population test coverage for women aged 23–60 years in Sweden 2014–2016.

County	Number of cytologies in 2014	Proportion of tests taken in the organized programme (%), 2014	Population Test coverage^1^, women 23–60 years in 2014 (%)	Number of cytologies in 2015	Proportion of tests taken in the organized programme (%), 2015	Population Test coverage, women 23–60 years in 2015 (%)	Number of cytologies in 2016	Proportion of tests taken in the organized programme (%), 2016	Population Test coverage, women 23–60 years in 2016 (%)
Stockholm	144,067	68	74	152,734	71	74	153,192	73	75
Uppsala	26,785	69	72	27,198	67	75	27,287	64	78
Södermanland	15,394	69	83	18,648	76	81	17,141	77	80
Östergötland	31,093	65	80	31,474	70	81	33,216	73	84
Jönköping	21,149	71	85	19,649	92	87	21,566	76	84
Kronoberg	9,137	80	71	11,124	70	69	10,881	71	70
Kalmar	13,581	72	83	17,077	77	81	15,305	73	84
Gotland	3,697	66	78	3,829	67	80	3,700	69	81
Blekinge	10,495	78	83	9,207	75	84	10,314	75	85
Skåne	89,076	55	78	101,689	59	81	100,384	53	82
Halland	22,399	66	90	22,475	68	90	21,973	70	90
Västra Götaland	122,487	73	89	125,096	70	89	126,849	66	88
Värmland	18,256	74	89	21,201	74	90	23,480	68	90
Örebro	18,112	78	82	21,507	75	84	21,648	77	85
Västmanland	16,721	75	83	15,903	72	84	15,859	72	82
Dalarna	20,470	75	91	20,736	72	91	17,860	76	92
Gävleborg	19,631	75	87	20,847	74	88	21,373	76	89
Västernorrland	15,611	75	85	13,266	72	81	20,511	83	86
Jämtland	8,252	73	87	7,980	71	86	8,785	70	87
Västerbotten	19,205	76	83	17,892	72	83	17,405	71	84
Norrbotten	16,732	74	84	16,116	74	85	14,217	72	84
**Sweden**	**662,350**	**69**	**81**	**695,648**	**70**	**82**	**702,946**	**69**	**82**

1) Population test coverage: The proportion of the population targeted by the screening program that has actually taken the test.

**Table 2:** Number of invited women 23–60 years in Sweden 2013–2015, and attendance rates within 3 months and 1 year.

		% of invited women with a cervical smear			% of invited women with a cervical smear			% of invited women with a cervical smear	
County	Number of women invited 2013	Within 3 months	Within 1 year	Number of women invited 2014	Within 3 months	Within 1 year	Number of women invited 2015	Within 3 months	Within 1 year
Stockholm	88,041	39	52	101,627	40	54	114,000	42	57
Uppsala	11,513	30	50	11,424	28	47	16,044	33	55
Södermanland	12,492	63	76	10,626	55	74	13,691	64	79
Östergötland	15,784	42	62	15,467	47	62	16,645	54	67
Jönköping	17,787	67	79	17,665	69	82	16,897	65	78
Kronoberg	9,087	39	59	7,681	38	57	7,844	33	56
Kalmar	7,332	62	71	6,975	67	75	9,851	71	78
Gotland	2,081	50	62	1,936	51	63	2,281	52	65
Blekinge	7,873	67	80	7,838	70	84	7,527	67	82
Skåne	55,288	44	62	54,306	73	85	13,832	78	87
Halland	16,095	77	87	12,474	64	78	64,053	53	66
Västra Götaland	76,095	67	76	80,410	50	64	83,666	66	75
Värmland	8,059	51	75	10,740	59	71	14,876	73	83
Örebro	12,122	59	72	13,067	53	67	13,029	66	76
Västmanland	10,743	60	74	12,118	69	77	11,239	60	77
Dalarna	10,626	64	80	13,131	69	81	12,787	69	83
Gävleborg	13,606	69	78	13,300	61	73	12,307	70	80
Västernorrland	12,480	73	78	11,790	63	78	8,604	68	74
Jämtland	5,296	65	74	5,554	66	80	5,402	66	76
Västerbotten	12,543	70	74	12,576	70	79	11,741	73	79
Norrbotten	10,969	65	80	8,929	70	77	10,750	63	77
**Sweden**	**415,912**	**55**	**68**	**429,634**	**68**	**77**	**467,066**	**57**	**70**

Analyses per age group are presented at www.nkcx.se_en. For example, the population test coverage in 2014 was 91.3, 80.3, 79.3, 79.5 and 82.7 percent for the age groups 23–25, 26–30, 31–40, 41–50 and 51–60, respectively. In 2015, the corresponding coverages were 91.9, 81.5, 79.6, 79.5, and 83.0 percent. Further, in 2016 the corresponding coverages were 90.3, 83.6, 80.4, 79.9, and 83.8 percent.

There were 7,982, 8,573, and 9,170 women with high-grade lesions in cytology in 2013–2015. Of these, 181, 279 and 228 women had not been followed up with biopsy by the end of the following year (as these analyses required follow-up, the period of the index smear ends the previous year). The proportion of women with histological follow-up within 3 months varied from 24 to 96% between counties and years, while the variation at 12 months only varied marginally (between 92–100%) (Table 3).

**Table 3:** Numbers of women diagnosed with HSIL+/AIS in 2013–2015, the cumulative proportion of HSIL+AIS in cytology that were followed with a biopsy anywhere in the country, within 3 month and within 1 year and women with HSIL+/AIS not followed up at the end of the following year.

		% of women followed up with histology				% of women followed up with histology				% of women followed up with histology		
County	Number of women with HSIL+/AIS in cytology, 2013	Within 3 months	Within 1 year	Number of women not followed up by 31-12-2014	Number of women with HSIL+/AIS in cytology, 2014	Within 3 months	Within 1 year	Number of women not followed up by 31-12-2015	Number of women with HSIL+/AIS in cytology, 2015	Within 3 months	Within 1 year	Number of women not followed up by 31-12-2016
Stockholm	1,679	84	96	48	1,729	76	90	123	1,904	86	97	32
Uppsala	107	83	94	4	144	83	97	2	230	80	97	4
Södermanland	223	55	96	4	130	60	92	5	161	78	96	7
Östergötland	316	85	98	3	415	84	99	2	422	84	99	2
Jönköping	353	86	98	4	360	72	98	5	209	59	96	7
Kronoberg	40	45	100	0	135	41	91	3	137	53	91	3
Kalmar	219	76	97	5	168	77	98	2	270	81	100	0
Gotland	74	96	99	0	66	85	95	3	54	79	90	4
Blekinge	146	57	97	3	224	67	99	3	252	64	97	4
Skåne	1,377	76	97	37	1312	76	96	40	1,382	55	93	72
Halland	238	74	97	4	243	77	98	4	219	70	95	5
Västra Götaland	1,533	66	96	40	1,562	64	95	52	1,707	57	96	37
Värmland	166	86	97	3	304	73	98	5	400	66	97	6
Örebro	241	73	97	3	377	66	97	7	297	54	96	7
Västmanland	123	80	98	3	148	88	100	0	185	81	96	7
Dalarna	130	72	98	2	190	90	98	4	178	74	96	5
Gävleborg	152	74	97	1	123	79	97	2	123	72	97	4
Västernorrland	189	54	98	3	244	46	97	2	209	50	97	5
Jämtland	119	41	98	1	139	35	98	2	124	24	99	1
Västerbotten	422	51	96	11	397	43	96	9	469	42	96	10
Norrbotten	135	66	97	2	163	66	97	4	238	70	97	6
**Sweden**	**7,982**	**71**	**97**	**181**	**8,573**	**70**	**96**	**279**	**9,170**	**66**	**96**	**228**

Thus, the key quality indicators showed either improving values or no change (Tables 1–3), which is in contrast to the data on cervical cancer incidence (below).

Around 90% of the cytologies were normal (e.g. 89–92% of cytologies taken in 2015 were normal). The proportion of smears with atypical squamous cells of undetermined significance (ASCUS), cervical intraepithelial neoplasia (CIN) 1 and CIN 2 (low-grade squamous intraepithelial lesion (LSIL)) diagnoses in cytology remained about the same during 2014–2016. In 2014, 4.8%, 2.6%, and 0.9%, in 2015 4.6%, 2.7% and 0.9%, and in 2016 4.9%, 2.8% and 0.9% of the smears respectively diagnosis and year, as did CIN3 (HSIL) in cytology (0.4% of smears in 2014, 0.5% of smears in 2015, and 0.5% of smears in 2016).

For comparison with the purpose of the program (to control cervical cancer), we retrieved the national age-standardized incidence of cervical cancer from national statistics. We found that it increased over time from 9.6 per 100,000 in 2014, (varying from 5.6 to 16.4 between counties) to 10.4 per 100,000 in 2015, (varying from 4.7 to 16.3 between counties) and 11.5 per 100,000 in 2016, (varying from 9.4 to 17.0 between counties) (p for trend (Wald test) = 0.03). Poisson regression comparing the 2014/2015 cervical cancer incidence with the incidence 2002–2013 as baseline found an 18% increase (RR: 1.18, 95% CI: 1.11–1.26, p <0.0001). In all previous years, there was either no trend or a decreasing trend. NKCx publishes full reports each year at http://www.nkcx.se (in Swedish).

## Discussion

This report describes the exact extent of cervical screening in Sweden during 2014–2016, such as the results of all the 2,278,000 cervical smears taken in this period and the proportion of the target population covered by the screening test (cytology or HPV testing) (81–82%). Key quality indicators are also reported for each county. Although the reasons for changes occurring over time in different counties, it is notable, that open publishing of data appears to influence the regionally organized programs. For example, the total number of women invited to screening in Sweden increased from 415,912 in 2013 to 467,066 women in 2015. This increase occurred concomitantly with our introduction of population invitation coverage (proportion of the population that does receive an invitation divided by the total population of women that should have received an invitation, according to the guidelines) as a publicly reported quality indicator.

Similarly, the reasons for the sometimes very large changes in population test coverage over time in different counties are not exactly known, but public reporting of low coverage’s has tended to result in increases.

Limitations of this evaluation is that data on other risk factors for cervical cancer, such as smoking et c, are not registered and could conceivably confound the analyses. The registry covers all real-life data in Sweden, but in the comparisons made the subjects are not randomized which could result in misleading conclusions. Furthermore, in early years of operation of the registry several labs exported codes that could not be interpreted. This has improved and today mostly interpretable codes are found. The existence of non-interpretable codes in earlier data could result in a bias in over-time analyses, but as these non-interpretable codes were found for only 0,36% of women (data posted at www.nkcx.se) any bias is not likely to be large.

Cervical screening is a globally recommended public health policy [6]. Although most European countries have mass screening registries where all cervical smears taken are registered, many of these registries do not report their annual analyses of the data in the scientific literature [1, 7]. Such reporting is essential to enable exchange of experiences, encouraging best practices and to provide an evidence base for innovation and improvements of the program. For example, the Swedish cervical screening registry is introducing quality indicators related to HPV-based screening. The new Swedish cervical screening guidelines mandate switching to HPV-based testing, at the following intervals: 3-yearly cytology in the ages 23–29, 3-yearly HPV testing in the ages 30–50, and a co-test with HPV and cytology at age 41, HPV testing every seventh year in the ages 51–64 [8]. Measures to ensure quality of the HPV testing itself include external proficiency panels [9] and laboratory audits of HPV analyses [10].The ability to monitor the impact of the policy change by performing registry-based follow-up of quality indicators based on comprehensive, individual level data was cited as an important consideration when the new program with HPV-based screening was recommended [8].

The increase in cervical cancer is both unexpected (there has been no previous increases for 50 years) and unexplained (no quality indicators have suggested a deterioration of quality that could explain the increase in the disease). The switch to HPV-based screening occurred too recently to have affected the incidence by increased detection of prevalent cases. An exploration of the registry data to search for possible explanations has recently been completed and reports that there is no increase among non-attending women, only among women attending and having normal smears, if analyzed at certain laboratories (other laboratories did not have this increase in cancer risk among women with normal smears) [11]. This suggests that additional quality indicators should be regularly measured and reported, as quality indicators should preferably herald if the cancer control is effective or not–already before changes in cancer incidence are seen.

## Conclusions

In summary, we find that comprehensive collection of all individual-level data on HPV tests, cytologies, histopathologies and invitations in a country are readily performed and that linkage of these data to calculate established quality indicators is straightforward and that a continuous improvement is seen in several quality indicators. However, the concomitant increase in cervical cancer suggests that current efforts for measuring and reporting quality indicators is insufficient.
